# The varied restorative values of campus landscapes to students’ well-being: evidence from a Chinese University

**DOI:** 10.1186/s12889-024-17952-w

**Published:** 2024-02-16

**Authors:** Xuanyi Nie, Yifei Wang, Chan Zhang, Yu Zhao, Niall Kirkwood

**Affiliations:** 1 Department of Urban and Regional Planning, School of Architecture and Planning, University at Buffalo, Buffalo, NY 14214 USA; 2 Harvard T.H. Chan School of Public Health, Boston, MA 02115 USA; 3 ScenesLab, Boston, USA; 4 College of Media and International Culture, Zhejiang University, Hangzhou, 310058 Zhejiang China; 5 College of Humanities, Tarim University, Alar, 843300 Xinjiang China; 6 Harvard University Graduate School of Design, Cambridge, MA 02138 USA

**Keywords:** Landscape, Restorative effects, Well-being, Student, University campus

## Abstract

**Background:**

The literature on therapeutic landscapes highlights that the university campus landscape has restorative effects on students. This deserves more scholarly attention since mental health has become an important issue among university students. However, existing empirical studies have revealed mixed evidence with little attention to the heterogeneity across the design and, therefore, the potential therapeutic effects across different landscapes.

**Method:**

This research examined how 13 landscape sites on a university campus might be differentially related to student well-being. These sites were identified from a variety of sources (campus design documents, photos used in the university’s social media posts, and interviews with a small group of students) to represent a comprehensive list of places that students might visit. The data was collected in a large online survey of a Chinese university (*n* = 2,528). We asked about students’ use of individual landscape sites and the associated motivations for visits, and measured well-being using a perceived stress scale and overall evaluation of the happiness level. Bivariate analysis was used to explore the zero-order associations between landscape use and well-being. OLS (for stress) and logistic regressions (for happiness) were conducted to further evaluate the associations after controlling the student background variables and potential correlations of uses across different landscapes.

**Results:**

Among 13 landscape sites, four sites had significant positive associations with either or both measures of well-being after controlling for the student characteristics and use of the other landscape sites. There was also an additive benefit of visiting more landscapes. Compared to those who did not frequently visit any of the sites, well-being had a significant stepwise increase among those who frequently visited one or two and more sites. One site that was significantly related to both measures of well-being only offered distant views of landscapes, but it was right next to the study areas.

**Conclusions:**

This study demonstrates the heterogeneity of restorative effects across different landscapes on campus. The findings suggest that effective landscape design that aims to promote student well-being should be placed close to stressors (i.e., where they study), and between where they study and live to offer students opportunities to break from the common routines and to relax. The findings hold greater relevance for universities in China and institutions with similar student campus lifestyles, occupancies, and behavior patterns worldwide.

**Supplementary Information:**

The online version contains supplementary material available at 10.1186/s12889-024-17952-w.

## Introduction

Landscape is an integral part of university campus designs. Landscape elements on university campuses not only introduce a sense of nature but also provide open spaces for student interactions among each other or with natural environments, which provide them with more pleasurable experiences [1, 2]. Based on that, scholars have identified the restorative effects of campus landscapes on students’ well-being, an increasingly important scholarly focus in environmental planning [3–8]. Restorative effects denote positive changes in psychological states [9], of which perceived stress and happiness are two fundamental elements [6, 7, 10, 11]. University campus landscapes can provide students with natural amenities and offer them spaces for restorative activities such as recreational sports and extracurricular clubs [12, 13].

The theory of therapeutic landscape has important implications for the restorative effect of the natural landscape [14]. This concept incorporates both aesthetic and more imperceptible social qualities of the landscape that connect humans and nature [15]. The aesthetic qualities are represented by the “biophilia hypothesis,” which argues that humans have spent almost all of their evolutionary history in the natural environment [16–19]. As a result, people are happier in natural habitats than in urban settings [20].

The social qualities are embedded in the transactions between a person and their broader socioenvironmental setting [21–23]. Such a relationship could be interpreted behaviorally through the provision of opportunities for individuals to engage in physical activities in natural environments [24]. In particular, the “enabling places” [21] acknowledge that the natural environment promotes physical activities that enable psychological regeneration. The “affective sanctuaries” highlight the importance of “third places” in the therapeutic landscapes [25], described as a retreat away from home (the first place) or work (the second place) [26]. This kind of “third places” can provide elusive opportunities for emotional refuge such as a feeling of being away from daily stress and a non-demanding social interaction recovery [4].

Existing studies have examined the impact of natural environments on campus (broadly defined as indoor and outdoor nature, as well as nature views) on student well-being, academic performance, as well as outcomes related to possible explanatory pathways (e.g., perceived restoration, temperature, physical behavior, etc.) [27]. Yet these studies showed mixed results. On the one hand, many studies suggest a positive relationship between university campus landscape and students’ well-being. Studies using data collected from universities in the United States, Scotland, and Turkey found that students with higher objective or perceived campus greenness in universities reported greater quality of life [7, 28]. Adding to them, another study in the United States concluded that only students who frequently engage with green spaces in active ways report higher quality of life, better overall mood, and lower perceived stress [29].

Meanwhile, some studies also report no substantial associations between campus landscape and students’ well-being. For example, a study in Austria yielded no significant correlation between perceived greenness and physical activity, a crucial determinant for physical health [30]. Although a study in the United States initially showed that students who use campus landscape more often reported higher quality of life [31], the follow-up work found that such a relationship is only valid for undergraduate students, not for graduate students [32].

The inclusiveness of the findings is unsurprising given the limited number of studies available and the heterogeneity in the study designs. In particular, the mixed results may be due to the heterogeneity of landscapes examined in the studies. Yet very few empirical studies have focused on comparing the restorative effects across landscapes. In the literature on the therapeutic landscape, landscape type is predominantly defined by a palette approach, categorizing landscape elements into “blue spaces” such as lakes and ponds, and “green spaces” including forests, grass, fields, and meadows [33–37]. Among the studies that specifically focus on university campuses, they tend to focus on single landscape sites or measure the overall levels of greenness [4, 5, 7, 28], overlooking the heterogeneity across campus landscape sites and its implications to students as the users.

This ignorance of nuances across different landscapes makes it difficult to develop practical guidance on how to design campus landscapes to promote student well-being. This is because existing studies suggest not all landscape sites are the same. For example, studies conducted in the United States, Spain, and China suggest that varied degrees of campus biodiversity [6], engagement with different activities [8, 38], and different types of green spaces [3] can yield different levels of restorative effects on students. These studies suggest the importance of considering heterogeneous landscapes when examining their influences on human behavior and well-being. However, these studies treated “landscape” homogenously as green spaces or natural elements, ignoring that landscape on a university campus broadly encompasses other types of open spaces such as paved areas with green elements or artificially design promenades that are proximate to natural elements.

Furthermore, previous research has not explicitly assessed whether there may be additive effects across exposure to different landscapes. University campuses usually include a range of landscapes spread across campus – near academic buildings, in the central area with common student resources such as the library, and at peripheral locations [7]. Students tend to use and appreciate campus green spaces and consider them essential elements of the campus environment [39]. With many kinds of natural environments found on university campuses, there may be various cumulative opportunities for restoration via potential interactions with campus landscapes at different distances and locations [40].

The potential therapeutic value of campus landscape seems particularly pivotal against the backdrop of rising mental health and well-being issues among university students. Based on the national surveys of undergraduate and graduate students in the U.S., the Healthy Minds Study finds that the percentage of students who met the criteria for one or more mental health problems increased from around 40% to more than 60% from 2013 to 2021 [41]. The high prevalence of health problems among university students is not unique to one country. A multi-nation survey in 2021 found alarming levels of high stress and depression among universities across countries during the coronavirus disease (COVID-19) pandemic [42].

Furthermore, although the international literature is growing, empirical research on the therapeutic values of the campus landscape in China is still very thin [43, 44]. Attention to this specific area becomes increasingly pertinent in light of China’s recent endeavors to promote “green universities” [45, 46]. Existing studies found that similar to the studies in the United States and other countries [7, 28, 29], the objective perception of the quality of landscapes or “naturalness” [47–49] and the capacity of providing spaces for students to relax [3] are positive factors to students’ restorative experiences and well-being. Although the results of these studies were promising, they shared the same limitations as we pointed out earlier – i.e., a decontextualized approach that fails to capture landscape heterogeneity and additive effects of restorative environment that are derived from chronic or repeated contacts with nature [50–52].

In response to the above gaps in the literature, this study investigates the relationship between campus landscape and students’ well-being in a major public university in Eastern China. In response to the above-mentioned limitations, this study aims to add to the existing literature by addressing the following research questions: (1) how does the impact on student well-being vary across different landscape sites on a university campus? (2) are there additive benefits across multiple landscape sites? (i.e., does visiting more landscape sites lead to a further increase in well-being?) We conducted a survey that included questions about how students use a variety of landscape sites on campus, which are designated outdoor spaces by design for student recreational and leisure uses. By simultaneously investigating the additive effect of a range of landscape sites on a Chinese university campus, we aim to provide a more comprehensive view of the role of campus landscapes in student well-being. Yet we also acknowledge that this study is conducted only at one university, thus the results may not be multipliable to other universities, for example, in different climates or cultural contexts where campus culture and students’ behavioral patterns differ.

## Materials and methods

### Data collection and participants

An online survey was conducted in June of 2021 among students at Zhejiang University in China. Ethical approval was granted by the Interdisciplinary Social Science Research Centre at Zhejiang University (Project ID: 202103–01). The items analyzed in this study were embedded in the longer questionnaire with a wide range of topics (e.g., experiences on campus, attitudes towards the university, etc.) and the questions on well-being and landscape were presented in different sections of the survey. Therefore, the aim of this study (i.e., evaluating the effect of landscape on well-being) was likely blind to the students, which helped reduce the potential bias in respondent compositions because of the topic interests. All the second-year students (*N* = 5,707) were invited to participate in the survey. The survey link was sent to students via their class "groups" on the SM platform (mainly WeChat or DingTalk) by the administration staff. The online questionnaire was programmed using the platform provided by SurveyPlus (https://www.surveyplus.cn/). If students accessed the questionnaire on a mobile device, the platform seamlessly redirected them to the mobile-friendly version.

### Well-being measures: perceived stress and happiness

Happiness [53] and stress [54] are commonly used well-being measures. Specifically, we used the 10-item version of the Perceived Stress Scale (PSS-10) [55]. The ten-item version has higher reliability and validity than other versions of the scale [56], with a validated Chinese translation of the questions [57]. We also conducted cognitive interviews with two undergraduates to check the validity of the Chinese version in our target population, and we made minor modifications to improve readability and clarity. For the happiness question, we adopted the single-item measure from the Chinese General Social Survey that asks respondents about their overall happiness levels, with a scale from 1 (feeling a lot of unhappiness) to 5 (feeling a lot of happiness). The question wording (in both English and Chinese) can be found in Additional file 1: Appendix A.

### Landscape site visits

**Fig. 1:**
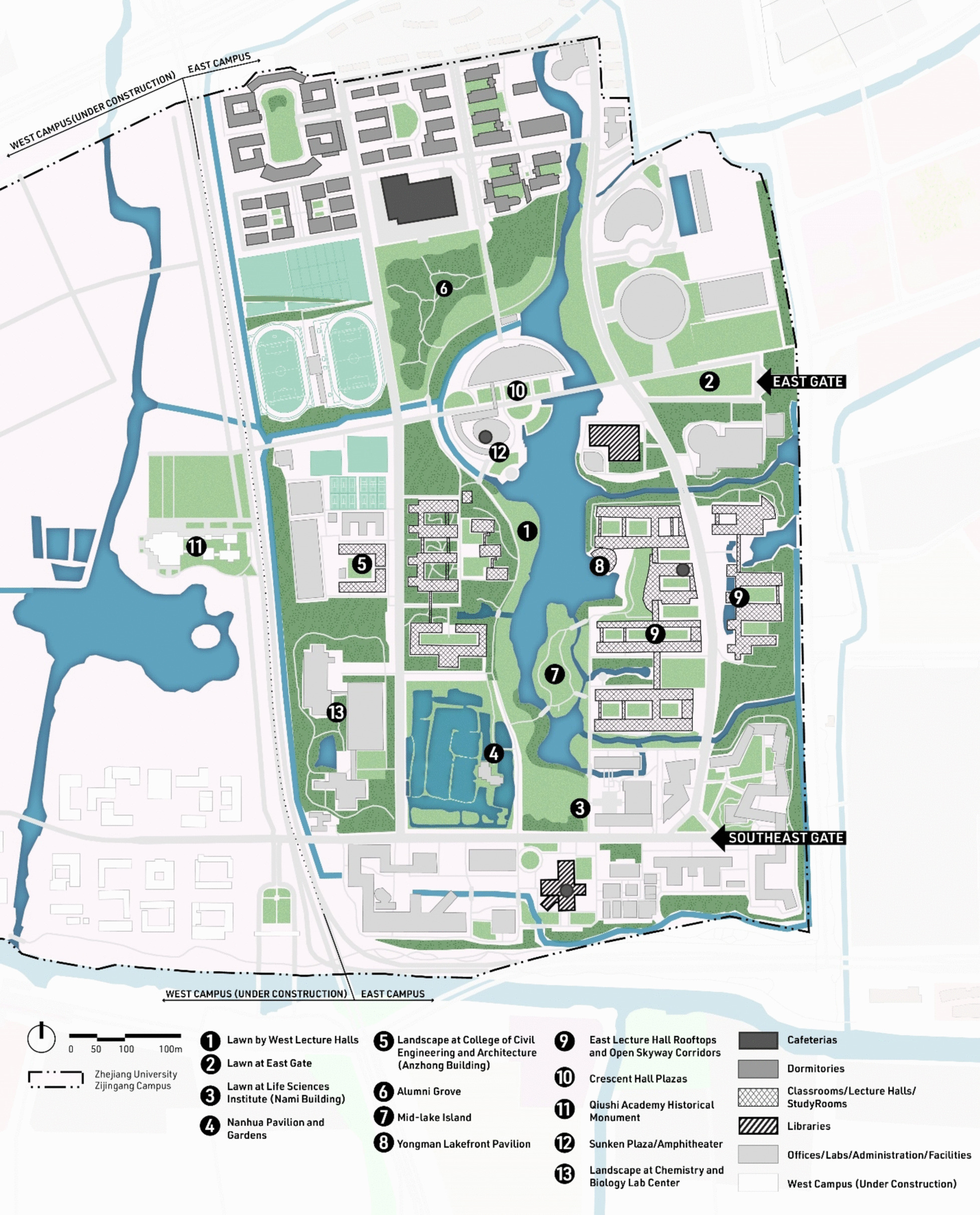
The locations of landscape sites on campus

We aimed to compile a comprehensive list of landscape sites that students might use on campus. We started with the campus design documents and excluded those of which the construction had not been completed by the time of this study. We then expanded the list by identifying additional sites that were featured in the university’s social media posts or mentioned in the interviews of a small group of students. This yielded a total of 13 landscape sites on the campus. The landscape sites are designated outdoor spaces by design for student recreational and leisure uses. They share common features such as seating elements, tree canopies, pedestrian pathways and trails, vegetation elements, and open green spaces for both passive and active uses. Some sites feature water attractions, gazebos, and seasonal and cultural elements. The 13 sites are distributed across the living compounds, classrooms, academic facilities, and main pathways, making them easily accessible. Figure 1 details the locations of the 13 landscape sites on the campus map. Please see Additional file 1: Appendix B for the photos and descriptions of each site.

In addition, students may use landscape sites differently according to their spatial or green qualities [8, 29]. We asked students whether they frequently visited the landscapes to gather information on the behavioral patterns of students on each site. When respondents reported frequently visiting a site, we had a follow-up question asking their reason(s) for visiting it, with the options including "party/team building," "study," "relaxation," "dating," and "group discussion," "dining," "exercising," and "being close to nature."

### Demographic variables

To control for the potential confounders (i.e., student characteristics that might be related to both landscape use and wellbeing), we collected in the survey various background information about the students, including gender, GPA, ethnicity, annual family income levels, highest education levels achieved by each parent, the province they were from, *Hukou* (household registration) status before college (urban vs. rural), and whether the students were in a romantic relationship or not. These variables were selected because existing literature has suggested that they might be related to students’ well-being. For example, an earlier study of this student population has found that stress was significantly related to gender, parents’ education, and family income (Tibber et al., 2023). Studies of the Chinese population in general have found urban hukou was associated with higher well-being than rural hukou (e.g., Tani, 2017). Besides, romantic relationships could either positively or negatively affect students’ well-being [58, 59]. However, our survey did not capture students’ majors or degrees, which could also be important influencers of their psychological state and behaviors.

### Analysis

We first conducted univariate analyses to show the distributions of student background characteristics, their happiness and stress levels, as well as the overall use of individual landscape sites on campus and the associated reasons. Then, we performed bivariate analyses to explore the zero-order associations between the number of landscape sites visited frequently and levels of well-being. Next, we used regression models to investigate whether such additive effects of landscapes held after controlling for various student background characteristics. Then, we treated the use of each landscape site as an independent variable and explored which site(s) promoted well-being after further considering the use of other landscape sites on campus. We used OLS regressions to model stress. For happiness, we first ran the ordinal logistic regressions and found that the proportional odds assumption did not hold for all the predictors. Therefore, we recoded the happiness variable into two categories (1 = a lot of unhappiness/some unhappiness/neither, 0 = a lot of happiness/some happiness) and used logistic regressions to model the likelihood of being in low levels of happiness.

## Empirical results

### Descriptive statistics

A total of 2,528 students completed the survey, with a response rate of 44.3%. Slightly more than half (54.8%) of the respondents were male. Among all the respondents, 44.2% of them had GPAs greater than 4 on a 0–5 scale, and approximately a quarter (25.6%) of them had GPAs below 3.5, with the rest (30.2%) in between. A total of 23.9% of the respondents reported being in a relationship. Most of the respondents were Han Chinese (92.7%). Approximately 70% of the respondents had urban Hukou (household registration) before being admitted to the college. Approximately half of the students (51.8%) were from Zhejiang Province.

Regarding socioeconomic status, the respondents contained a mix of students from different backgrounds. Approximately one-third of the students had an annual family income of over 200,000 CNY (Chinese yuan) (approximately 30,000 USD). Another 30% of the students had a family income between 100,000–200,000 CNY, with the rest split between the two lowest income categories (< 50,000 CNY: 16.7%; 50,000–100,000 CNY: 17.5%). We classified parents' education levels into three categories: middle school or less, high school or equivalent, and bachelor's degree or more. For mothers' education levels, the percentage of respondents in each category was 31.6%, 41.9%, and 26.6%, respectively. The fathers' education showed a similar distribution with overall slightly higher levels than the mothers' education. (For the regression analyses below, we only used the mother's education as the predictor, as the education levels of both parents were correlated.) See the upper part of Table 1 for the detailed distributions of respondents' demographics.

**Table 1:** Univariate analysis of respondent demographics and well-being measures

Variable	Percentage
**Demographics**	
Gender	
Male	54.8%
Female	45.2%
GPA	
> 4.00	44.2%
3.50–3.99	30.2%
< 3.49	25.6%
Ethnicity	
Han	92.7%
Minorities	7.3%
Province	
Zhejiang	51.8%
Other	48.2%
Hukou registration	
Rural	31.4%
Urban	68.6%
Family Income	
< 50 k CNY	16.7%
50 k ~ 100 k CNY	17.5%
100 k ~ 200 k CNY	30.4%
> 200 k CNY	35.4%
Mother's education	
Middle school or less	31.6%
High school or equivalent	41.9%
BA or more	26.6%
Father's education	
Middle school or less	26.2%
High school or equivalent	38.4%
BA or more	35.4%
In a relationship	
Yes	23.9%
No	76.1%
**Well-being**	
Happiness	
A lot of unhappiness	1.5%
Some unhappiness	4.8%
Neither	30.1%
Some happiness	51.9%
A lot of happiness	11.6%
Stress	
Mean	27.61
Sd	6.52
Min	10
Max	50

For happiness, a total of 11.6% of the respondents chose the highest category (i.e., "feeling a lot of happiness"), 51.9% of them reported feeling some happiness, 30.1% chose the middle category indicating they were between happiness or unhappiness, and very few of them chose the two negative options ("feeling some unhappiness": 4.8%; "feeling a lot of unhappiness": 1.5%). For the PSS-10, the Cronbach’s Coefficient Alpha is 0.89. In the confirmatory factor analysis that specified a one-factor model, the model fit indices were acceptable. Specifically, the comparative fit index (CFI) was 0.83; the Tucker-Lewis index (TLI) was 0.78; the root mean square error of approximation (RMSEA) was 0.16. We calculated the total score from the PSS-10 after recoding the four reverse-worded items so that for each item, higher scores indicated a higher level of stress. The average score for stress is 27.6 (min = 10 and max = 50) with a standard deviation of 6.5 (See the lower part of Table 1 for the detailed distributions of respondents' well-being measures).

Table 2 shows the percentage of frequently visiting each landscape site and the corresponding reasons for landscape visits. Not all landscape sites were visited equally. The most popular site was site #1 (Lawn by West Lecture Halls), and 51.8% of the respondents reported visiting it frequently. The second tier included four sites, which were Mid-lake Island (#7), Crescent Hall Plazas (#10), Sunken Plaza/Amphitheater (#12), and Alumni Grove (#6), ordered by popularity, with the percentages of frequent visits ranging between 20 and 30%. The rest of the sites had fewer than 20% of the respondents who reported visiting them frequently. We also checked the correlations of visiting these sites (see details in Additional file 1: Appendix C). Most of the correlations were around or below 0.3, except for Sites #10 and #12 (*r* = 0.48). This is expected because the two sites, being adjacent to each other on either side of a street (as can be seen in Fig. 1), share a cohesive design approach with similar characteristics and site features.

**Table 2:**
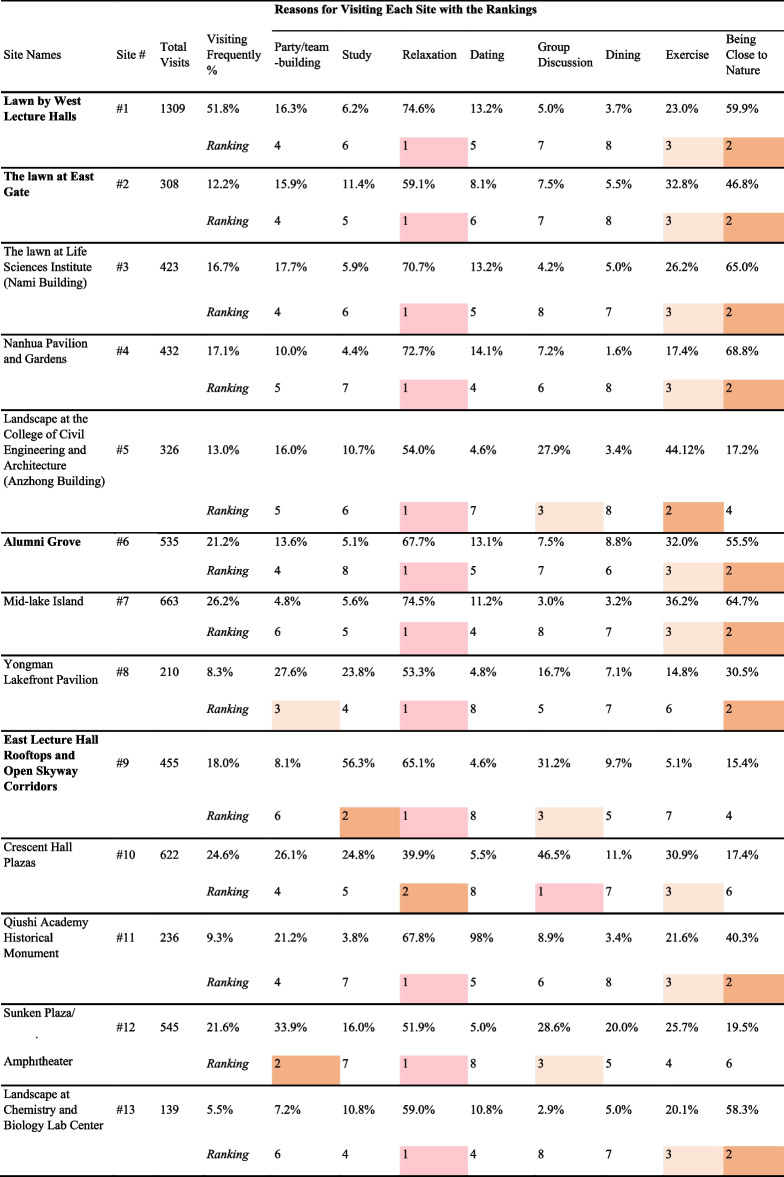
Percentage of frequently visiting each landscape site and the corresponding reasons (top three most selected reasons were shaded for each site)

The colors annotate the top three reasons for using the landscape sites; the bold texts in Site Names annotate the landscape sites that were significantly related to well-being in the regression analyses as shown in Table 3

Table 2 also shows the reasons for visiting each site, “relaxation” was the most mentioned reason for most of the sites, “being close to nature” was ranked next for most of the sites, and “exercise” was often the third most mentioned reason. While most of the sites have “relaxation”, “exercise”, and “being close to nature” as the top three reasons, Site #9 (East Lecture Hall Open Skyway Corridors) is distinctive from the others in that the majority (56.3%) of the respondents reported visiting it for studying, a reason much less mentioned for other sites.

We also calculated the total number of sites they reported frequently visiting for each respondent. 29.5% of the students reported not frequently visiting any of the sites. The percentages of frequently visited one and two sites were 15.9% and 15.3%, respectively. The number was reduced to 13.1% for visiting three sites, and it started to drop more quickly from there. To have adequate sample sizes to represent different levels of landscape visits, we recoded this variable into three categories (0, 1–2, and 3 or more sites) for the analyses after.

### Bivariate analysis

**Fig. 2:**
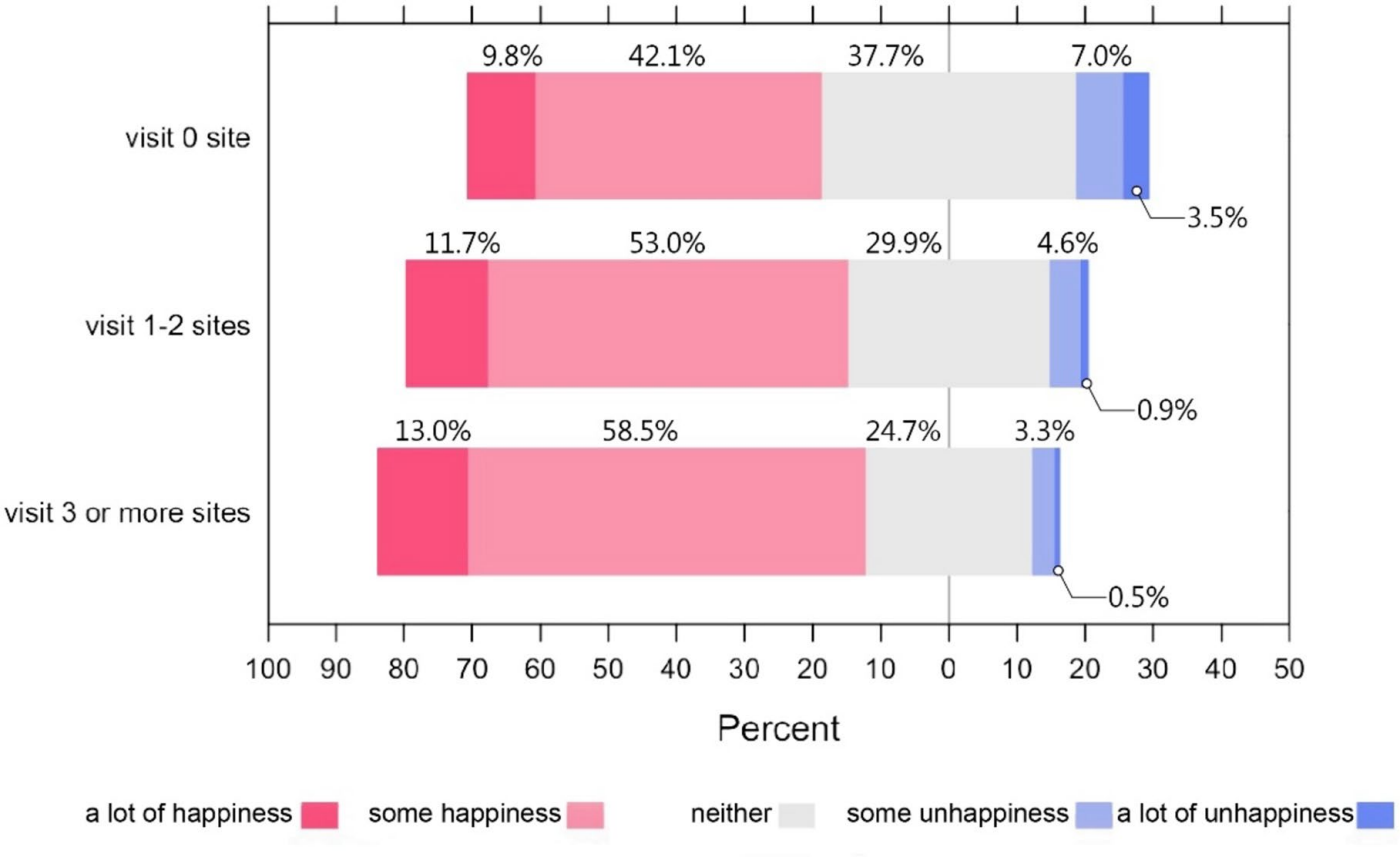
The distribution of respondents' reported happiness and frequency of site visiting

**Fig. 3:**
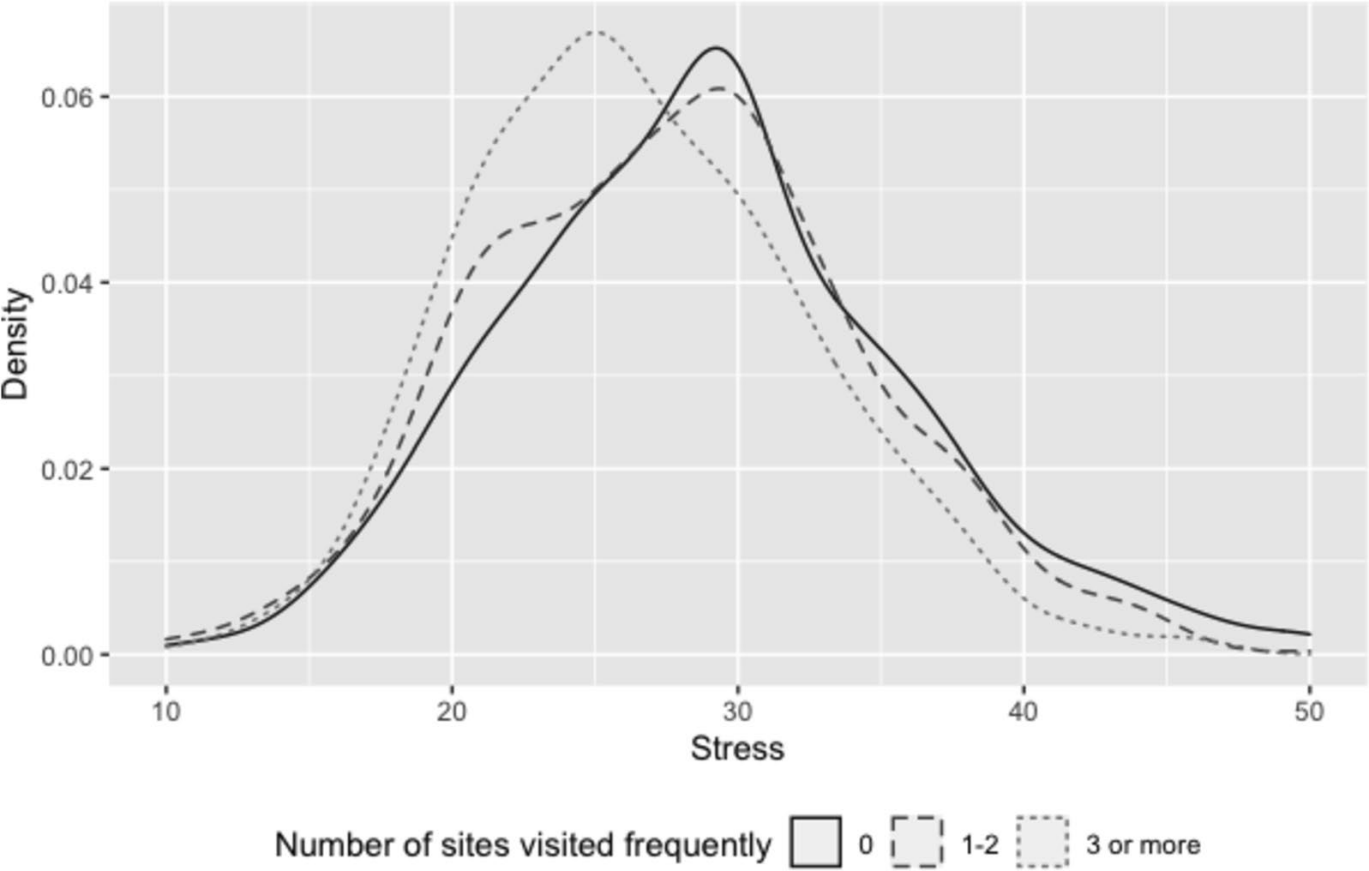
Distribution of stress and frequency of site-visiting

Figure 2 shows the distribution of the happiness answers by three levels of landscape visits (none, 1–2, and 3 or more sites). As students frequently visited more landscape sites, the distributions of the answers to the happiness question shifted toward the positive side, and this association was significant. A chi-square test of independence revealed a significant association between students’ levels of happiness (low = a lot of unhappiness/some unhappiness/neither; high = a lot of happiness/some happiness) and three levels of landscape visits (${\chi }^{2}(2)$=71.19, *p* < 0.001). Figure 3 compares the distributions of stress for students with three different levels of landscape visits. The distributions of stress appear to shift toward lower ends for the students visiting more landscape sites. The average stress scores were 28.8, 27.8, and 26.5 for the three groups (from the highest landscape visits to the lowest), respectively. The ANOVA test showed that the differences in stress across these three groups were significant, *F*(2, 2496) = 26.84, *p* < 0.001. (See Additional file 1: Appendix D for the bivariate analysis of key student demographics and two well-being measures.)

### Regression analyses

Following our objectives, we tried two approaches to depict landscape visits in association with stress and happiness in the regression analyses: (1) a categorical variable indicating the overall level of landscape visits (i.e., frequently visiting 0, 1–2, and 3 or more sites) and (2) dummy variables for frequently visiting individual sites. The first approach allows us to examine whether there is an additive effect of visiting different landscape sites on well-being. The second approach enables us to assess whether the impact on well-being is the same across various landscape sites.

The regression results are reported in Table 3. In response to our objective of examining the additive effects of landscape sites, compared to the students who did not frequently visit any of the landscape sites, those who frequently visited 1–2 sites and 3 or more sites had significantly lower levels of stress (1–2 sites: coeff. = -1.33, se = 0.38, *p* < 0.001; 3 or more sites: coeff. = -2.43, se = 0.36, *p* < 0.001). A further test shows that these two coefficients were significantly different (*t* = 3.20, *p* = 0.001), suggesting that frequently visiting more landscape visits has additive effects of reducing stress levels (see Model 1 in Table 3). Essentially, the same pattern was found with happiness (Model 3), with frequently visiting landscape sites associated with a lower likelihood of being in a lower level of happiness compared to not frequently visiting any of the sites (1–2 site, coeff. = -0.48, se = 0.11, *p* < 0.001; 3 or more sites: coeff. = -0.68, se = 0.11, *p* < 0.001). The difference in the effects between visiting 1–2 sites and 3 or more sites was also significant (*z* = -2.07, *p* = 0.039), suggesting that frequently visiting more sites is associated with an even lower likelihood of reporting lower levels of happiness.

**Table 3:** Results of the OLS (Stress) and logistical (Happiness) regressions

Variables	Stress		Low level of happiness^a^	
	Model1	Model2	Model3	Model4
Male (vs. female)	-1.294***	-1.279***	0.106	0.145
	[0.299]	[0.299]	[0.100]	[0.100]
Han Chinese (vs. minorities)	0.062	0.074	0.112	0.066
	[0.556]	[0.554]	[0.183]	[0.183]
Mother's education (ref = middle school or less)				
High school or equivalent	0.177	0.100	0.092	0.076
	[0.396]	[0.395]	[0.129]	[0.129]
BA or more	-0.141	-0.195	-0.237	-0.247
	[0.479]	[0.478]	[0.161]	[0.160]
Annual family income (ref = 50 k CNY or less)				
50 k-100 k CNY	-0.021	0.043	-0.221	-0.190
	[0.499]	[0.497]	[0.161]	[0.161]
100 k-200 k CNY	-0.345	-0.353	-0.330*	-0.330*
	[0.473]	[0.472]	[0.154]	[0.154]
200 k CNY or more	-0.913	-0.937	-0.250	-0.262
	[0.492]	[0.491]	[0.161]	[0.161]
GPA (ref = 3.5 or lower)				
3.5–4.0	-0.169**	-1.165**	-0.204	-0.193
	[0.395]	[0.394]	[0.129]	[0.129]
4.0 or higher	-1.200**	-1.187**	-0.294*	-0.282*
	[0.375]	[0.374]	[0.123]	[0.123]
Zhejiang Province (vs. other provinces)	-0.377	-0.361	-0.323**	-0.312**
	[0.318]	[0.316]	[0.106]	[0.106]
Households registered as rural (vs. urban)	0.049	0.029	0.155	0.150
	[0.388]	[0.387]	[0.127]	[0.127]
In a relationship (vs. not)	-0.278	-0.256	-0.414***	-0.441***
	[0.339]	[0.337]	[0.117]	[0.117]
Level of landscape use (ref = frequently visit zero sites)				
Frequently visit 1–2 sites	-1.330***		-0.522***	
	[0.375]		[0.121]	
Frequently visit 3 sites or more	-2.430***		-0.768***	
	[0.360]		[0.119]	
Individual site				
#1		-1.461**		-0.377***
		[0.310]		[0.102]
#2		–		-0.398*
		–		[0.161]
#6		-1.053**		–
		[0.377]		–
#9		-1.017**		-0.414**
		[0.389]		[0.138]

^a^For happiness, logistic regressions were used to model the likelihood of being in the low level of happiness (a lot of unhappiness/some unhappiness/neither) as opposed to being in the high level (a lot of happiness/some happiness). Standard errors are in brackets. * *p* < 0.05, ** *p* < 0.01, *** *p* < 0.001

Responding to our objective of examining the varying restorative effects across different landscape sites, we found that frequently visiting sites #1 (lawn by West Lecture Halls) and #9 (East Lecture Hall Open Skyway Corridors) were significantly associated with both lower levels of stress and a lower likelihood of being at lower levels of happiness. As shown in the univariate analyses, site #1 was the most popular site on campus. Site #9 was also unique because "study" was mentioned much more often as the reason for visiting it than reported for the other sites. In addition to those two sites, frequently visiting Site #2 (lawn at East Gate) was associated with a lower likelihood of reporting lower levels of happiness, and Site #6 (Alumni Grove) was significantly associated with lower levels of stress. The effects of the other sites were not significant after we controlled for respondent demographics and the aforementioned sites (see Models 2 and 4 in Table 3 for the details of the individual effects of different sites).

Lastly, some of the sociodemographic characteristics of students are significantly related to their well-being. Female students, compared to male students, reported significantly higher levels of stress, with no significant gender difference for happiness. A very significant predictor for student well-being, whose dominant pressure comes from academic performance, was GPA. Having a higher GPA was significantly related to experiencing less stress and feeling more happiness. Meanwhile, being a romantic relationship was significantly associated with more happiness, although it was not significantly correlated with their perceived stress.

## Discussion

Our results suggest that the campus landscape on a university campus could increase happiness and reduce stress. This conforms with the previous studies on the restorative values of university campus landscape elements [5, 7, 28, 60–62]. On top of that, our empirical analysis highlights the varying importance of the human well-being of different types of campus landscapes. We examined 13 sites with different locational features concerning the entire university campus.

Among the 13 sites, sites #1, #6, and #9 are significantly associated with the reduction of stress, and sites #1, #2, and #9 are significantly associated with happiness. This suggests that the restorative value of landscape sites functions differently across different sites, which complements the previous studies on university campus landscapes which only focus on single sites or treated multiple sites homogenously as green open spaces [4, 5, 7, 28]. In addition, we found significant increases in well-being across the students who reported frequently visiting 0, 1–2 sites, and 3 or more landscape sites on campus. The restorative effect of the campus landscape is thereby additive.

Whilst we made no a priori predictions as to what sort of landscape would be associated with higher levels of well-being, we are able to make some tentative post hoc speculations by drawing on the theories of the therapeutic landscape. Site #1, #2, and #6 all feature vegetation and water elements, which are important facets of “biophilia" [16–19]. However, we also found that the biophilic design itself is not sufficient in explaining the heterogeneity among the identified landscape sites. The relationship between landscape sites and stress reduction or happiness should also be integrated with the relational sociological approaches of “enabling spaces” [21] and "affective sanctuaries" [25].

Among the 13 landscape sites, Sites #1, #4, #7, #8, #12, and #13 feature water bodies and waterfront designs. However, only Site #1 is identified as being associated with stress reduction and more happiness. Through the principles of “enabling spaces” [21], Site #1 is directly located next to the lecture hall building cluster adjacent to the water features. While the vegetation and water body could provide students with a sense of connection with nature, the site also provides students with space for socialization because students use the lecture halls often. Table 2 also shows that Site #1 is the most accessed landscape site among all the 13 sites. This suggests that a landscape site needs to be frequently accessed to be able to perform restoratively for students. Furthermore, most students go there for relaxation (74.6%), being close to nature (59.9%), and exercise (23.0%), which aligns with principles of biophilia and enabling space.

Site #2 is an open lawn with no shade or trails, accompanying the East Gate to signify a grand entry. Site #1, #2, and #3 all feature open lawns. Site #2 is associated with happiness but not stress reduction. Our explanation is coined to the “symbolic meaning” of the site. Although it does not provide an intercept between study and living, this site may signify symbolic values of taking a break from the university thus escaping to “affective sanctuaries”, which might help contribute to higher levels of happiness [63, 64]. Similar to Site #1, most students go there for relaxation (59.1%), being close to nature (46.8%), and exercise (32.8%), presenting the significance of biophilia and enabling space.

Site #6 is a densely planted grove between campus living districts (cafeteria and dormitories) and academic districts (libraries, labs, lecture halls). Aside from its cultivated repertoire of trees and colors that feature biophilic qualities, it can serve as the affective sanctuary between the first place of academic districts and the second place of campus living districts, offering a temporary break from students' daily routines [4, 25, 26]. This suggests that landscape spaces are more restorative when disseminated along or between the daily paths that students tend to take. Like Sites #1 and #2, most students go there for relaxation (67.7%), being close to nature (55.5%), and exercise (32.0%), highlighting the functions of this site in biophilia and enabling space.

A majority (56.3%) of the respondents reported visiting site #9 for "studying," a reason much less mentioned for the other sites. Furthermore, site #9 is not a space for students to be out there in nature, it is a rooftop area attached to and connected between classroom buildings. A series of shaded open corridors offers students a good distant view of the campus lake, lawn, canopy, and other open spaces. This conforms with the studies on views of nature and restoration that even looking at images of nature has a positive impact on emotional and physical responses to stressors [9, 65]. The results about Site #9 conform with a previous study which suggests that visual connections with landscape elements are restorative [61]. In our research, Site #9 suggests that instead of physically being in the space, visual connections to open space and ground landscape have restorative effects.

Overall, we found three of the four landscape sites with significant positive associations with well-being tend to be located close to where students would spend most of their time. Table 2 shows that Site #1 is the most frequently visited (51.8%), and the visiting frequency of Site #2 is 12.2%, 21.2% for #6, and 18.0% for #9. The pattern is less clear as to how other designs of landscape might be related to the effectiveness in promoting well-being. For example, site #1 has a lakefront, but there are other sites with water elements that have no significant associations with well-being. Similarly, several sites feature a large open lawn, but not all of them are positively related to well-being. This suggests that simply categorizing various landscapes based on a list of vegetation types or “colors” might not be informative of their potential restorative value. The location, which denotes the frequency of visits and the “get away” function of landscape sites, and the spatial organization, which determines the types of activities that could happen in landscape sites may point to a more profound direction to understand the relationship between campus landscape and students’ well-being.

We acknowledge that this study has several limitations. Our data was collected from a cross-sectional survey. Thus, the findings regarding the relationships between landscape visits and well-being were correlational, not causal. Additionally, this research was carried out on one university campus located in East China where the local climate features warmth and humidity. Such climatic conditions and the corresponding landscape features may influence how students engage with the landscapes. The findings might be different for universities situated in regions with colder and drier weather. Moreover, the sample in this study only included second-year students. The findings might be different for other student groups. For instance, among third- or fourth-year students, who often experience heightened stress due to job searches or thesis preparation, the benefits of being exposed to the campus landscape might be more pronounced. Finally, our measurement of landscape use was based on students’ self-report of whether they frequently visited a landscape site, and terms such as “visit” and “frequently” may be subject to individual interpretation. Due to the lack of data on the exact frequency and duration of landscape visits, this study was unable to address the question concerning the levels of landscape exposure required to yield restorative effects. Future research should aim to establish causal evidence using longitudinal data with more objective and compressive measures of landscape visits (e.g., GPS data indicating the duration of stay) across different university campuses and diverse student populations.

## Conclusion

This research empirically examines the association between campus landscape and students' well-being in China through the lens of the therapeutic landscape. The results confirm the positive relationship between the two fields [5, 7, 28, 60–62]. Our research adds to the existing studies by pointing out two implications. Firstly, not all landscape sites are the same in psychological restoration. A campus landscape seems to function more effectively when it is located closer to sources of academic pressures such as lecture halls and classrooms or along students' daily commuting routes, which provides a break from students’ everyday routines. Secondly, the restorative value of the campus landscape is positively associated with more landscape visits. Even visual linkages with nature have a positive impact on emotional and physical responses to stressors [9, 65].

Together, our findings complement the existing literature on the therapeutic landscape and university campuses. This study sheds light on important implications for future campus planning and design in China. More calibrated design strategies could help promote the campus landscape’s restorative capacity for students. Existing campuses can also seek opportunities to repurpose adjacent underutilized lots, converting them into landscape interest to provide direct access or sensory (such as visual) interest for vital improvements. In addition, Chinese university campuses are typically situated at a distance from urban centers and are enclosed. Students' daily activities are predominantly concentrated on campus, frequent at locations integral to their daily routines, such as attending classes, studying, and dining. Notably, the campus culture in China entails students' devoting a considerable amount of their time outside of class to studying in designated areas, typically unoccupied classrooms. This is a unique aspect of campus life in China, closely related to the concentration of stressors. The findings of this study hold greater relevance for universities in China and institutions with similar student campus lifestyles, occupancies, and behavior patterns worldwide.

### Supplementary Information

**Additional file 1: Appendix A.** Question wording for measures of happiness and stress. **Appendix B.** Site description. **Appendix C.** Correlation matrix of frequently visiting different landscape sites. **Appendix D.** Bivariate analysis of key demographics and two well-being measures.
