# Supplementary material for: The varied restorative values of campus landscapes to students’ well-being: evidence from a Chinese University
Source: BMC Public Health. 2024 Feb 16;24:487. doi: 10.1186/s12889-024-17952-w (PMC10870671; doi:10.1186/s12889-024-17952-w)
Supplement: Supplementary file 1 — Additional file 1: Appendix A. Question wording for measures of happiness and stress. Appendix B. Site description. Appendix C. Correlation matrix of frequently visiting different landscape sites. Appendix D. Bivariate analysis of key demographics and two well-being measures. [file 12889_2024_17952_MOESM1_ESM.docx]

**Appendix A. Question Wording for Measures of Happiness and Stress**

| Variable | Items (English) | Items (Chinese) |
| --- | --- | --- |
| Happiness | Overall, do you feel happiness in your life?  (A lot of unhappiness/some unhappiness/neither/some happiness/a lot of happiness) | 总的来说，您觉得您的生活是否幸福?  (非常不幸福/比较不幸福/说不上幸福不幸福/比较幸福/非常幸福) |
| 10-PSS | How often have you …?  1. been upset because of something that happened unexpectedly?  2. felt that you were unable to control the important things in your life?  3. felt nervous and “stressed”?  4. felt confident about your ability to handle your personal problems?  5. felt that things were going your way?  6. found that you could not cope with all the things that you had to do?  7. been able to control irritations in your life?  8. felt that you were on top of things?  9. been angered because of things that happened that were outside of your control?  10. felt difficulties were piling up so high that you could not overcome with?  (never/almost never/sometimes/fairly often/very often) | 下列情况是否符合您的实际情况：  1.因为一些意外发生的事情而感到沮丧  2.对生活中重要的事情感到无法控制  3.感到紧张和焦虑  4.有信心自己能够处理好自己的问题  5.感到事情顺心如意  6.发现自己难以应对所有不得不做的事情  7.能够控制生活中让人恼怒的事情  8.感到事情在自己的掌控之中  9.因为事情超出自己的控制而生气  10,棘手的问题越来越多以至于你觉得自己无法解决  （从不/偶尔/有时/常常/总是） |

**Appendix B. Site Description**


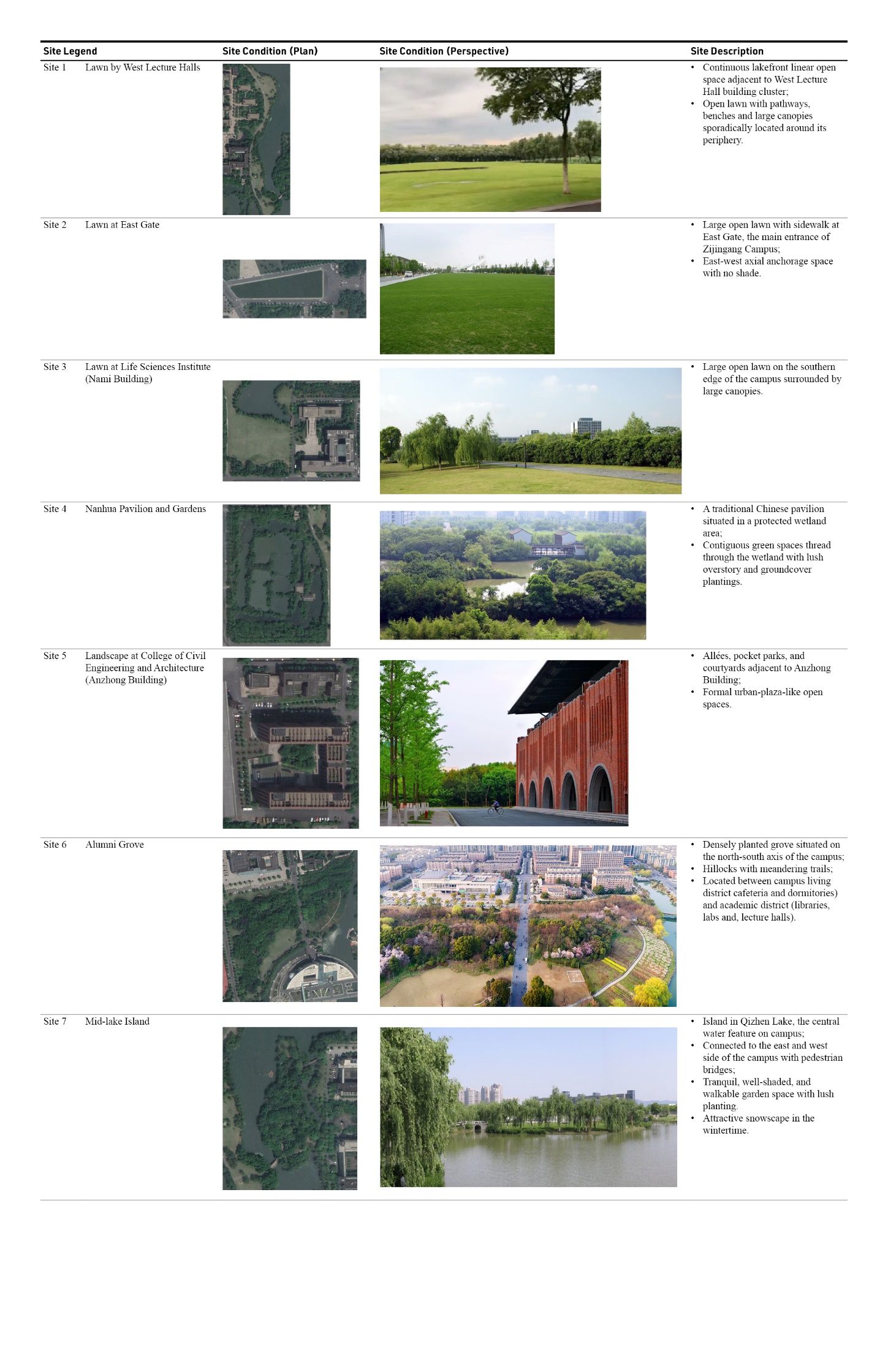


**Appendix B. Site Description (Continued)**


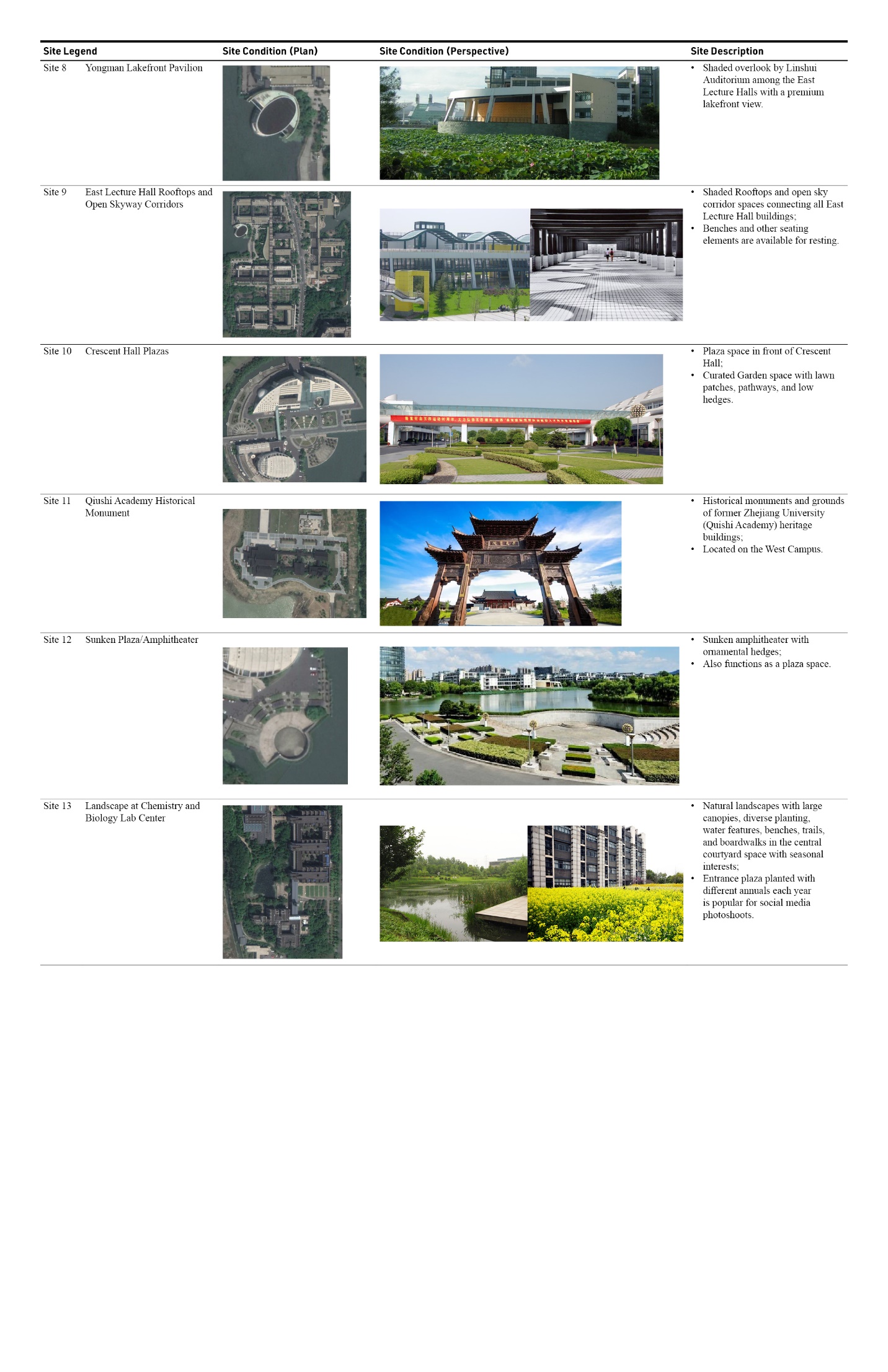


**Appendix C. Correlation Matrix of Frequently Visiting Different Landscape Sites**

|  | Site 1 | Site 2 | Site 3 | Site 4 | Site 5 | Site 6 | Site 7 | Site 8 | Site 9 | Site 10 | Site 11 | Site 12 | Site 13 |
| --- | --- | --- | --- | --- | --- | --- | --- | --- | --- | --- | --- | --- | --- |
| Site 1 | 1.000 |  |  |  |  |  |  |  |  |  |  |  |  |
| Site 2 | 0.236 | 1.000 |  |  |  |  |  |  |  |  |  |  |  |
| Site 3 | 0.305 | 0.287 | 1.000 |  |  |  |  |  |  |  |  |  |  |
| Site 4 | 0.268 | 0.216 | 0.348 | 1.000 |  |  |  |  |  |  |  |  |  |
| Site 5 | 0.199 | 0.333 | 0.258 | 0.249 | 1.000 |  |  |  |  |  |  |  |  |
| Site 6 | 0.298 | 0.328 | 0.281 | 0.346 | 0.301 | 1.000 |  |  |  |  |  |  |  |
| Site 7 | 0.320 | 0.177 | 0.323 | 0.379 | 0.219 | 0.371 | 1.000 |  |  |  |  |  |  |
| Site 8 | 0.170 | 0.335 | 0.268 | 0.294 | 0.320 | 0.297 | 0.277 | 1.000 |  |  |  |  |  |
| Site 9 | 0.223 | 0.222 | 0.187 | 0.184 | 0.253 | 0.239 | 0.261 | 0.333 | 1.000 |  |  |  |  |
| Site 10 | 0.242 | 0.270 | 0.165 | 0.146 | 0.260 | 0.286 | 0.200 | 0.267 | 0.285 | 1.000 |  |  |  |
| Site 11 | 0.193 | 0.275 | 0.271 | 0.328 | 0.331 | 0.276 | 0.238 | 0.312 | 0.197 | 0.189 | 1.000 |  |  |
| Site 12 | 0.248 | 0.296 | 0.201 | 0.230 | 0.226 | 0.317 | 0.232 | 0.285 | 0.273 | 0.482 | 0.199 | 1.000 |  |
| Site 13 | 0.118 | 0.255 | 0.278 | 0.278 | 0.306 | 0.232 | 0.247 | 0.298 | 0.203 | 0.152 | 0.316 | 0.198 | 1.000 |

*Note.* The correlations reported above are Kendall’s rank correlations.

**Appendix D. Bivariate Analysis of Key Demographics and Two Well-being Measures**

| Variable | Stress | Low happiness (%) | Variable | Stress | Low happiness (%) |
| --- | --- | --- | --- | --- | --- |
| **Gender** | *F*(1,2497)=8.68,  *p*=0.003 | $\chi^{2}$(1)=8.23,  *p*=0.004 | **Mother's education** | *F*(2,2496)=1.024,  *p*=0.359 | $\chi^{2}$(2)=15.06,  *p*<0.001 |
| male | 27.26 | 39.0% | middle school or less | 27.68 | 40.0% |
| female | 28.04 | 33.4% | high school or equi | 27.75 | 37.5% |
| **GPA** | *F*(2,2496)=6.66,  *p*=0.001 | $\chi^{2}$(2)=24.57,  *p*<0.001 | BA or more | 27.31 | 30.5% |
| >4.00 | 27.26 | 32.1% | **In a relationship** | *F*(1,2497)=5.982,  *p*=0.015 | $\chi^{2}$(1)=25.61, *p*<0.001 |
| 3.50-3.99 | 27.45 | 36.4% | Yes | 27.04 | 27.7% |
| <3.49 | 28.41 | 43.9% | No | 27.79 | 39.2% |
| **Ethnicity** | *F*(1,2497)=1.65,  *p*=0.199 | $\chi^{2}$(1)=0.50,  *p*=0.478 | **Province** | *F*(1,2497)=1.142,  *p*=0.285 | $\chi^{2}$(1)=8.27,  *p*=0.004 |
| Han | 27.57 | 36.2% | Zhejiang | 27.48 | 33.7% |
| Minorities | 28.21 | 39.1% | Other | 27.76 | 39.3% |
| **Family Income** | *F*(3,1968)=3.37,  *p*=0.018 | $\chi^{2}$(3)=19.91,  *p*<0.001 | **Hukou registration** | *F*(1,2497)=0.731,  *p*=0.393 | $\chi^{2}$(1)=9.29,  *p*=0.002 |
| <50k CNY | 28.15 | 45.0% | Rural | 27.54 | 34.4% |
| 50k~100k CNY | 28.07 | 39.0% | Urban | 27.78 | 40.8% |
| 100k~200k CNY | 27.65 | 33.6% |  |  |  |
| >200k CNY | 27.00 | 31.9% |  |  |  |

*Note.* Low happiness means that for the happiness question, respondents reported “a lot of unhappiness”, “some unhappiness”, or “neither”
